# SARS-CoV-2 Drug-Resistant Mutations in Non-Structural Proteins

**DOI:** 10.3390/v18090993

**Published:** 2026-09-09

**Authors:** Madison Shaw, Anthony R. Fehr

**Affiliations:** Department of Molecular Biosciences, University of Kansas, Lawrence, KS 66045, USA

**Keywords:** coronavirus, antivirals, drug resistance, main protease, RNA-dependent RNA polymerase, papain-like protease, endoribonuclease, macrodomain

## Abstract

Various antiviral drug therapies have been developed for the treatment of coronaviruses, particularly SARS-CoV-2. However, the emergence of drug-resistant mutations is detrimental to clinical efficacy and global public health. Antiviral usage exerts selective pressures on viruses that manufacture an environment for resistant strains to emerge, in some cases at a fitness cost. However, the appearance of a compensatory mutation can restore or improve viral fitness, allowing the strain to persist and spread in a population. Here we evaluate the drug resistance mechanisms of multiple SARS-CoV-2 non-structural proteins, including the main protease (Mpro) and the RNA-dependent RNA polymerase (RdRP), which drive polyprotein processing and viral RNA replication, as well as PLPro, EndoU, and Mac1, which contribute to viral replication and counter host innate immune responses. We also discuss several methods that could be used to avoid drug resistance in the future. By integrating the understanding of molecular mechanisms of antiviral treatment with surveillance of resistance-associated mutations and new drug therapies, appropriate clinical approaches can be developed to reduce the impact of drug resistance.

## 1. Introduction

Coronaviruses are a family of positive-sense RNA viruses characterized by their surface spike glycoproteins, which resemble crown-like projections. Coronaviruses originated in bats but have evolved to infect a wide variety of both mammalian and avian species. In addition, there are seven known human coronaviruses [1]. The three coronaviruses that have caused the greatest severe illness in the population are SARS-CoV, MERS-CoV, and SARS-CoV-2. SARS-CoV-2 is the causative agent of COVID-19, a respiratory illness that includes symptoms such as fever, cough, shortness of breath, and dyspnea [2]. Clinical presentation ranges from mild to severe, and the virus transmits primarily through respiratory droplets and aerosolized particles [3]. Viral entry of SARS-CoV-2 occurs when the spike (S) protein binds the ACE2 receptor of host cells [2]. As the primary attachment protein, the spike protein is the main target for antibody neutralization [4]. The virus then enters the cell through membrane fusion or endocytosis, releasing the RNA genome into the cytoplasm to begin utilizing the host cellular machinery for viral replication [2]. The host ribosomes then translate the viral RNA into polyproteins, which are subsequently cleaved into functional non-structural proteins (nsps). The main protease (Mpro), encoded by nsp5, and the papain-like protease (PLpro), encoded as a domain of nsp3, mediate these cleavages. PLpro cleaves nsp1/2, nsp2/3, and nsp3/4, while Mpro cleaves all the remaining junctions. Several of the non-structural proteins form a replication–transcription complex (RTC), including nsps 7, 8, and 12, which form the core of the RTC, with nsp12 acting as the polymerase that copies the RNA genome and produces sub-genomic RNAs that act as templates for the production of structural and accessory genes. Several additional non-structural proteins are also part of the RTC and promote capping of viral mRNAs, provide proofreading capabilities, and protect the RNA from detection by RNA sensors, including MDA-5 [5]. The structural proteins are then translated, with the membrane-bound structural proteins being translated in the endoplasmic reticulum (ER). The structural proteins then traffic to the ER–Golgi intermediate compartment, where they interact with the N protein and genomic RNA to form mature virions. The mature virions egress through the ER–Golgi and are released from the cell using lysosomal exocytosis pathways to infect neighboring cells [6].

## 2. Coronavirus Antivirals

Various antiviral treatments have been investigated for the treatment of COVID-19, and several are currently approved for use (Table 1). Antivirals function to stop viral infections through mechanisms such as preventing entry through receptor inhibition, reducing viral RNA replication, or enabling host immune responses [7], which can reduce the severity of symptoms and shorten recovery time. SARS-CoV-2 has many conserved enzymatic processes that are vital for replication, making these enzymes potential antiviral drug targets. Targeting these replication machinery enzymes with antiviral drugs can repress viral replication. The first antiviral treatments investigated were nucleoside analogs such as remdesivir (Veklury^®^), which was originally developed by Gilead in search of hepatitis C virus (HCV) RdRP inhibitors [8], and molnupiravir (Lagevrio^®^), as well as protease inhibitors such as nirmatrelvir [9]. Remdesivir and molnupiravir target the RNA-dependent RNA polymerase (RdRp), or non-structural protein 12 (nsp12), and inhibit viral RNA transcription and replication. Perhaps the most widely used CoV antiviral is Paxlovid^®^ [10]. Paxlovid^®^ is the combination of nirmatrelvir, which targets the main protease (Mpro), and ritonavir, which acts as a pharmacokinetic enhancer by inhibiting cytochrome P450 3A (CYP3A), thereby slowing the metabolic degradation of nirmatrelvir and increasing its effectiveness [11]. Currently, Veklury^®^ and Paxlovid^®^ are the only FDA-approved antivirals for the treatment of SARS-CoV-2, while Lagevrio^®^ is permitted solely for emergency usage [9]. The antiviral ensitrelvir (Xocova^®^), which also targets the Mpro, is an approved treatment in Japan and Singapore and is currently under review by the FDA in the United States [12]. Additionally, several other viral non-structural proteins, including the papain-like protease (PLpro-nsp3) [13,14], the helicase (nsp13) [15], the macrodomain (Mac1-nsp3) [16], the endoribonuclease (EndoU-nsp15) [17] and the N- and O-methyltransferases (NMTase/OMTase-nsp14/16) [18,19] have also been investigated as a drug targets because of their crucial role in viral replication and host immune evasion, as well as their highly conserved active sites.

Even with the clinical success of these treatments, there is a rising concern about resistance with the widespread and long-term use of these medications. It is important to note that coronaviruses have a proofreading capability through the exonuclease ExoN, which reduces the overall mutation rate and also mediates resistance to nucleoside analogs such as remdesivir and molnupiravir [9]. However, the mutation rate for SARS-CoV-2 is about 1 in every 10^6^ nucleotides, or about 1 mutation in every 30 genomes produced, though this rate has changed throughout the pandemic [20]. Thus, these viruses are still able to evolve resistance under selective pressures from antiviral drugs, which allows for the emergence of drug-resistant strains [9]. Antiviral resistance arises through viral mutations fueled by high replication rates, host immune escape, incomplete suppression, and selective drug pressures [9]. The mutations can alter inter-host and intra-host interactions, influencing replication and transmission efficiency. While some mutations can enhance viral replication, they can also reduce transmission, demonstrating a fitness tradeoff where certain viral functions are improved to the detriment of others. Due to the tradeoff, many mutations do not lead to massive outbreaks because the strain’s overall fitness is decreased. However, if a compensatory mutation is also acquired that restores or enhances fitness, this can lead to the emergence of unique viral variants, similar to how novel variants emerged following vaccination [21]. This is termed epistasis. Understanding these interactions is critical for managing the global health impacts of viral variants.

## 3. Molecular Mechanisms of Resistance

### 3.1. Main Protease (Mpro)—(nsp5)

Mpro is a cysteine protease that cleaves viral polypeptides during replication [11]. This is an essential function of viral replication for the creation of functional proteins, which is why it is a major drug target and has led to the identification of several drug-resistant mutations (Table 2). Based on mutational analysis in cell culture, SARS-CoV-2 variants with Mpro mutations in the presence of nirmatrelvir revealed E166V, L27V, N142S, A173V, and Y154N to be among the most common resistance mutations, demonstrating how multiple mutations can confer resistance [22]. However, these mutations also showed decreased catalytic activity, which may explain why they have not been identified in sequences isolated from COVID-19 patients treated with Paxlovid. When evaluating mutations seen in the clinic, nirmatrelvir resistance has been observed when the A285V mutation co-occurs with P123H and K90R mutations [22]. In addition, a T21I mutation was observed in an immunocompromised individual, which conferred about a 4-fold increase in resistance to nirmatrelvir [23]. However, a number of mutations in Mpro have been identified in naturally occurring variants, likely in response to the use of Paxlovid in the clinic. The appearance of so many mutations may be due to routinely using the same protease inhibitor over several years.

**Figure 1:**
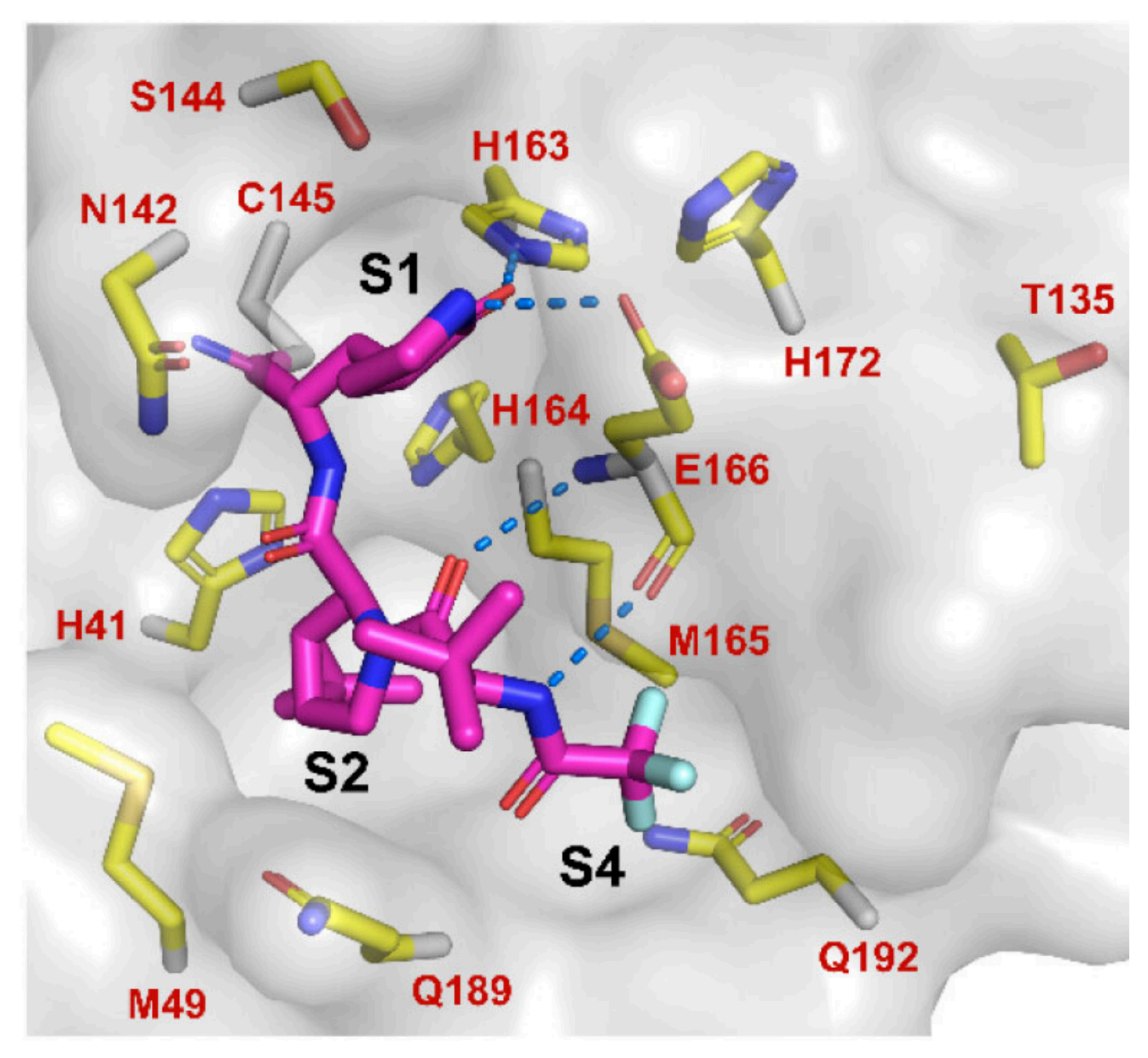
Mpro crystal structure highlighting nirmatrelvir binding and residues involved in drug resistance. Nirmatrelvir (magenta) and residues within 6 Å of the nirmatrelvir-binding site (yellow), including S144, M165, E166, H172, and Q192. This figure was generated with using the Pymol Molecular Graphics System (Schrödinger LLC, New York, NY, USA), displaying the X-ray crystal structure of nirmatrelvir in a complex with SARS-CoV-2 Mpro (PDB: 7SI9). Imaged obtained from Hu et al. [11] without modification. https://creativecommons.org/licenses/by/4.0/.

A set of mutations in the main protease sequence identified in naturally occurring variants, S144M/F/A/G/Y, M165Y/T, E166V/G/A, H172Q/F, and Q192T/S/L/A/I/P/H/V/W/C/F, showed comparable replicative capabilities while being resistant to nirmatrelvir [11]. Several of these residues are known to be critical for binding to nirmatrelvir, including M165, E166, and H172 (Figure 1). S144 is located in the S1 pocket in an oxyanion hole to stabilize enzyme function. Pfizer’s report on the S144A mutant stated that it caused a >90-fold increase in *K*_i_ (the inhibitor dissociation constant) [11]. This demonstrates the clinical relevance of the mutation, as it can reduce the drug’s binding affinity while remaining enzymatically active. The S144M/F/G/Y mutations also confer high resistance to nirmatrelvir while retaining enzymatic activity. M165 is in the S2 pocket, forming a hydrophobic interaction with P2 dimethylcycloproylproline [11]. The most common mutation at this residue was M165Y, but it was associated with decreased enzymatic activity. In contrast, the M165T mutant has significant drug resistance but no decrease in enzymatic activity, displaying a 29.9-fold increase in *K*_i_ [11]. E166 is in the S1 pocket and binds to nirmatrelvir through three hydrogen bonds [11]. E166G was found to be resistant to nirmatrelvir but with a 7.4-fold reduction in enzymatic activity. Several independent studies have found that the E166A and E166V mutants, isolated or co-occurring with other mutations, are nirmatrelvir-resistant and exhibit comparable enzymatic activity to WT protein [11,24]. H172 interacts with nirmatrelvir indirectly and is in the S1 pocket. The mutants H172Q and H172F both exhibited significant drug resistance with comparable enzymatic activity to WT protein [11]. Q192 is in the S4 pocket and interacts with the trifluoromethyl substitution from nirmatrelvir hydrophobically [11]. Q192T/L/I/P/H/V/W/C/F mutations all showed nirmatrelvir resistance but with enzymatic activity between 3.5- and 9.2-fold reduced when compared to WT protein.

Mpro is also the target of the drugs ensitrelvir, ibuzatrelvir, simnotrelvir, and bofutrelvir. A separate study of naturally occurring variants looking at resistance to both nirmatrelvir and ensitrelvir found that ΔP168, A173V, and A173T conferred resistance to nirmatrelvir, while T45I, D48Y, M49I/L/T/V, and ΔP168 conferred resistance to ensitrelvir. In addition, a number of combination mutants had substantially increased resistance to one or both inhibitors, including ΔP168/A17V, T45I/M49L, T45I/A173V, D48Y/ΔP168, M49I/ΔP168, and M49L/ΔP168 [4].

**Figure 2:**
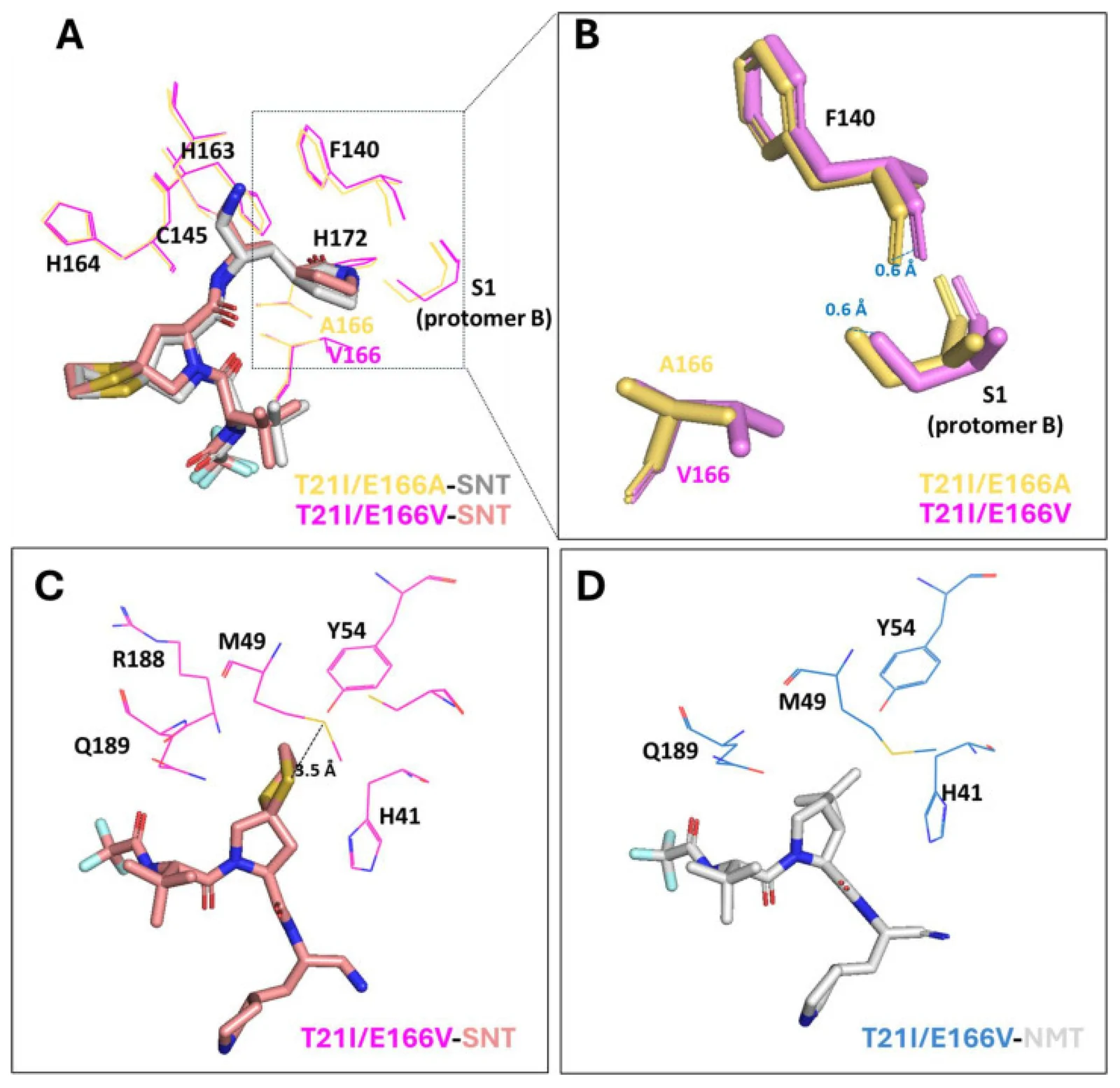
Binding modes of simnotrelvir (SNT) or nirmatrelvir (NMT) in combination with T21I/E166V or T21I/E166A mutations in the catalytic pockets of Mpro. (**A**) Overlay of the binding modes of SNT with T21I/E166V and T21I/E166A. Hydrogen and covalent bonds formed with F140, C145, H163, H164, H172, and Ser1 residues (protomer B) shown. (**B**) Detailed movements (blue dashed lines) of F140 and Ser1 residue impacted by valine substitution compared with alanine substitution. (**C**) Interactions of P2 segment of SNT and the S2 subsite residues of T21I/E166V. S-S interaction (black dashed line) at 3.5 Å is established between the two sulfur atoms. (**D**) P2 segment NMT and the S2 subsite residues of T21I/E166V. Image was obtained from Chen et al. [26] https://doi.org/10.1128/jvi.02223-25 without modification. https://creativecommons.org/licenses/by/4.0/.

In cell culture experiments, M49L and E166A substitutions in nsp5 are associated with increased resistance to ensitrelvir [25]. These mutations render the drug less effective while still maintaining viral replication and transmission efficiency. Specifically, the alanine substitution at E166 disrupts hydrogen bonds in the γ-lactam rings of ensitrelvir [26]. The M49L mutant was selected under ensitrelvir drug pressure due to a structural change in the S2 binding pocket that prevents the drug from binding [27]. However, structural modeling reveals that ibuzatrelvir contains many hydrophobic interactions in the S2 pocket, allowing it to remain active with the M49L mutation [27]. As M49L remains sensitive to this drug, ibuzatrelvir could be a treatment option if this mutation appears in patients, as it has occurred in natural variants of the virus [4,27]. The E166A mutation, while resistant to ensitrelvir, remained susceptible to nirmatrelvir, the only protease inhibitor currently used in the clinic. So, unless ensitrelvir replaces the use of nirmatrelvir in patients, this mutation is unlikely to appear in the clinic [25]. One of the major concerns moving forward is the possibility of multiple mutations or a single mutation that confers multidrug resistance, as has been observed with antibiotic resistance. The E166V mutation has been shown to confer broad-spectrum resistance to most protease inhibitors [27]. Much like the alanine mutation, the lack of hydrogen bonding, together with a conformational change, reduces binding to these inhibitors within the pocket [27]. The E166A mutation, in combination with the T21I substitution, which itself has led to resistance in the patients [23], has conferred a 16-fold to 30-fold increase in drug resistance to the protease inhibitors simnotrelvir, nirmatrelvir, and ensitrelvir, but not to bofutrelvir [26]. This is due to bofutrelvir forming a covalent bond and a distinct structure with these residues of Mpro. While the T21I mutation is distal to the active site, the E166A mutation has a significant impact on the S1 subsite through the disruption of a hydrogen bond [22]. This causes a conformational change by interrupting binding between protomer A and protomer B of the active site [22] (Figure 2). When considering positive epistasis, both the L232R and T21I mutations can restore enzyme activity to the E166V mutant Mpro in the presence of inhibitors [27]. These mutations can confer multidrug resistance to simnotrelvir, nirmatrelvir, and ensitrelvir while replicating normally in the absence of inhibitors, indicating that these mutations could emerge in a population of susceptible individuals [26].

**Table 2:** Mpro drug-resistant mutations discussed in this review.

Mutation	Drug	Reference
T21I	nirmatrelvir	[23]
L27V	nirmatrelvir	[22]
N142S	nirmatrelvir	[22]
A173V	nirmatrelvir	[22]
Y154N	nirmatrelvir	[22]
A285V + P123H + K90R	nirmatrelvir	[22]
E166V/G	nirmatrelvir, ensitrelvir, bofutrelvir	[11,24,25,27]
S144A/M/F/G/Y	nirmatrelvir	[11]
M165Y/T	nirmatrelvir	[11]
H172Q/F	nirmatrelvir	[11]
Q192T/L/I/P/H/V/W/C/F	nirmatrelvir	[11]
E166A E166V + T21I	ensitrelvir	[25]
	simnotrelvir, nirmatrelvir, and ensitrelvir	[27]
E166V + L232R	simnotrelvir, nirmatrelvir, and ensitrelvir	[27]
E166A + T21I	simnotrelvir, nirmatrelvir, and ensitrelvir	[27]
ΔP168	nirmatrelvir, ensitrelvir	[4]
A173V/T	nirmatrelvir	[4]
T45I	ensitrelvir	[4]
D48Y	ensitrelvir	[4]
M49I/L/T/V	ensitrelvir	[4,27]
ΔP168 + A17eV	nirmatrelvir and ensitrelvir	[4]
T45I + M49L	ensitrelvir	[4]
T45I + A173V	nirmatrelvir and ensitrelvir	[4]
D48Y + ΔP168	nirmatrelvir and ensitrelvir	[4]
M49I + ΔP168	nirmatrelvir and ensitrelvir	[4]
M49L + ΔP168	nirmatrelvir and ensitrelvir	[4]

### 3.2. RNA-Dependent RNA Polymerase (RdRp)—nsp12

**Figure 3:**
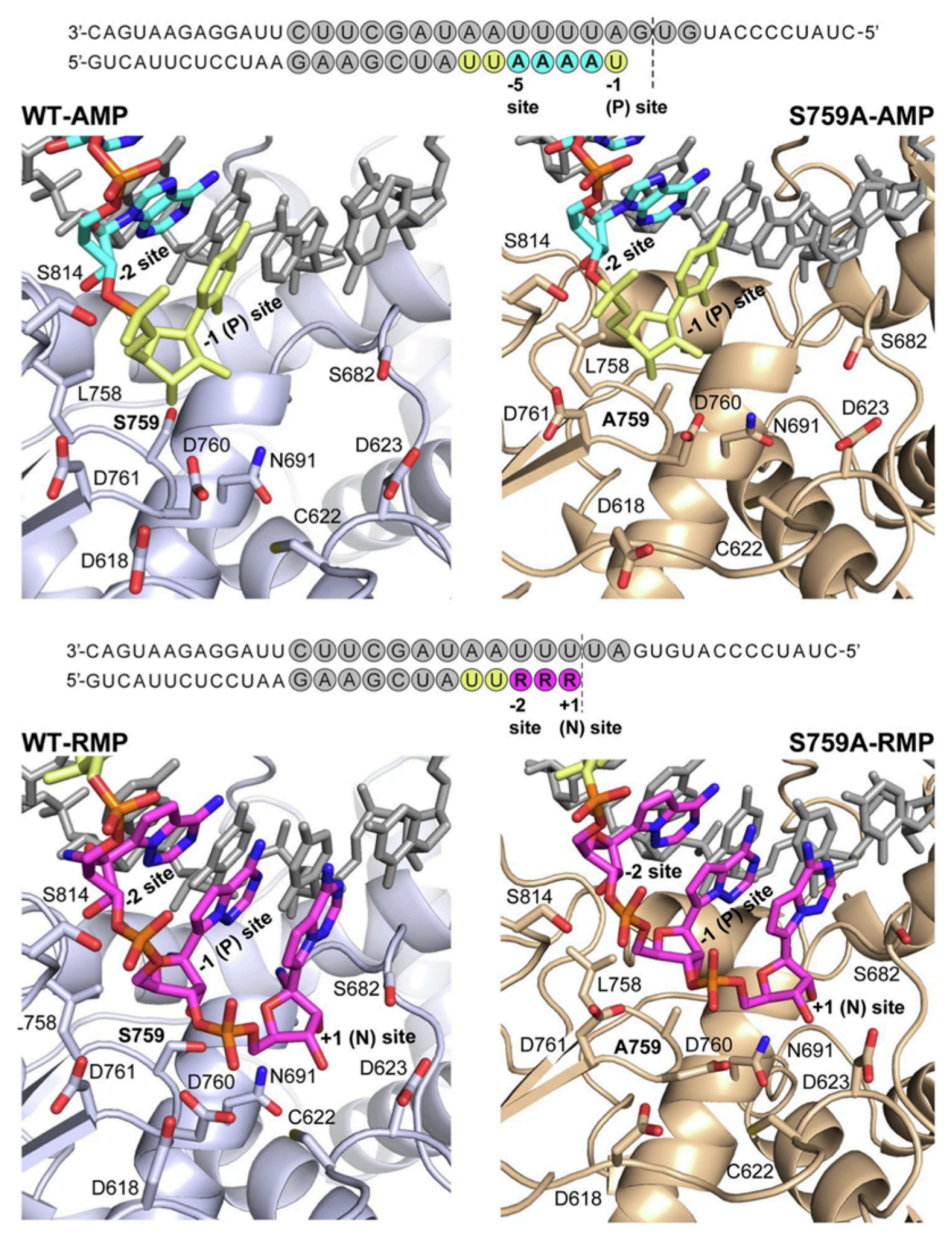
(**Top**) Schematics of WT (light purple) and S759A (gold) RdRp active site bound to AMP (cyan) is displayed. (**Bottom**) Interaction between WT and S759A RdRp active sites bound to RMP (magenta). The template and primer strand bases (gray) are incorporated into UMP (yellow) and AMP (cyan). Amino acid residues are shown as sticks, displaying favorable interactions. Imaged was obtained from Gordon et al. [31] https://doi.org/10.1038/s42003-026-09844-z without modification. https://creativecommons.org/licenses/by-nc-nd/4.0/deed.en.

The RNA-dependent RNA polymerase (RdRp) adds single nucleotides to elongating viral RNAs and is essential for viral replication [28]. Due to the highly conserved sequence of RdRp, this protein is a major target for drug development [9] and is the target of remdesivir [28]. Remdesivir acts as a nucleoside analog and terminates chain elongation, placing the polymerase under selective pressure, which could lead to resistant mutations. There are only a few nsp12 mutations that have occurred in the clinic, including an E802D mutation that occurred in an immunocompromised individual. This mutation resulted in a 6-fold increase in sensitivity to remdesivir but had a fitness cost associated with it [29]. Several other nsp12 mutations were observed in another immunosuppressed individual treated with remdesivir, including C799F, V792I, and M794I, with C799F being largely responsible for drug resistance. Experimental studies in laboratory conditions show that single-nucleotide polymorphisms in Nsp12, such as F480L, V557L, and V792I in RdRp, can lead to resistance to remdesivir [28,30]. In addition, an S759A mutant demonstrated antiviral resistance to remdesivir through a mechanism that altered the preference of the RdRp for ATP over RTP, the active form of remdesivir [30,31]. The S759A mutation alters the interactions between ring structures on RTP and incoming bases, reducing van der Waals interactions, while having minimal impact on adenine (from AMP) interactions with incoming bases (Figure 3). This results in ATP being incorporated into the S759A RdRp at a 10-fold excess in comparison with RTP, decreasing its antiviral activity [31]. Importantly, RdRp remdesivir-resistant mutants typically occur at a fitness cost. The F480L mutation destabilizes the interface between protein domains, the V557L affects the binding of the RNA to RdRp, while the S759A mutation disrupts polymerase efficiency in the active site through the loss of a hydrogen bond, inducing a conformational change [31]. These mutants showed reduced overall viral replication in the absence of remdesivir, indicating decreased fitness in the absence of the antiviral [28]. In addition, the S759A and V792I mutations were introduced into corresponding residues in MHV and were found to confer resistance to remdesivir but also had significant replication defects [30]. These findings are largely based on cell culture systems rather than clinical data, so their clinical relevance to global populations remains unclear. While resistance mutations for both RdRp and Mpro have been observed, Mpro has a greater potential for developing drug-resistant mutations than RdRp. The active site of Mpro has a higher mutational frequency, indicating less-rigid requirements for specific amino acids in the active site, which places it at risk for mutations that are able to resist antivirals. RdRp requires a more conserved sequence to remain functional [28,32]. Remdesivir-resistant escape mutations are therefore uncommon in comparison [28]. Regardless, in comparison with mutations of the spike protein that emerge in response to vaccination, there is a much lower chance of escape mutations in Mpro or RdRp enzymes [4]. In addition to viral targets of currently utilized therapeutics, several other viral proteins have been tested for drug resistance to early-stage inhibitors. Here we will highlight some of the drug resistance mutations that have been identified in three of these targets, the papain-like protease (PLPro—nsp3) the endoribonuclease (nsp15), and the macrodomain or Mac1 (nsp3), though additional resistance mutations in these or other targets may have been identified.

### 3.3. Endoribonuclease (EndoU)—nsp15

The nsp15 endoribonuclease (EndoU) is thought to aid in host immune evasion and the regulation of viral RNA synthesis [33]. Nsp15 EndoU avoids early induction of IFN-I through a reduction in viral dsRNA replication intermediates [33]. An inhibitor of nsp15, EPB-113, was developed to target an interferon-independent mechanism, ultimately selecting for escape mutants that lead to a replication defect. EPB-113 was shown to inhibit SARS-CoV-2 replication at an EC_90_ of 12 µM and produced more than a 5-log10 reduction at 50 µM [33]. Its antiviral effect is closely linked with nsp15 and the EndoU domain [33]. Residues G248 and H250 were found to be conserved at the catalytic center of EndoU [33]. Under selective pressure due to the presence of EPB-113, the mutations G248V and H250Y were found in the breakthrough viruses. EPB-113 was strongly affected by the resistant mutation T66I, with the EC_90_ value relative to WT being almost as pronounced (12-fold) as that of the G248V and H250Y mutants. However, a K60R mutant did not produce a significant shift in the EC_90_ value [33].

### 3.4. Papain-like Protease (PLpro)—nsp3

The papain-like protease (PLpro) is a globular domain of nsp3 involved in both the cleavage of nsps and the regulation of the innate immune system through the deconjugation of ubiquitin and ubiquitin-like molecules [34]. PLpro is able to retain proteolytic activity in the absence of nsp3 sequences, with involvement from the S4 and S3 pockets [34]. Although the active site is where the enzymatic reactions take place, inhibitors have been designed to target the S4 pocket [34]. The GRL0617 series of inhibitors, originally designed to target SARS-CoV, works by inhibiting through engagement of the blocking loop and occupation of the S4 pocket [34]. Although these inhibitors are not currently approved for clinical use, efforts to improve their fit in the S3 and S4 pockets could increase their inhibitory efficacy [34]. Two leading derivatives of this class, PF-07957472 and Jun12682, as well as a structurally distinct inhibitor, WEHI-P8, which binds to a similar adjacent pocket, have been analyzed for potential resistant hotspots [34]. Results from the biochemical assays showed that the mutations D164C and G266S affected all three compounds through a reduction in inhibition by 40–400 fold [34]. D164 participates in conserved hydrogen bonding, whereas G266 influences the flexibility of the blocking loop [34]. The mutations Y268R/C, found in the blocking loop, E167G, and Q269H strongly affect PF-07957472 and Jun12682 inhibition but only mildly affect WEHI-P8 inhibition [34]. WEHI-P8 activity is selectively reduced by the M208W, N267K, and T301A mutations, altering interactions in the pockets, the blocking loop, and indirect inhibitor stabilization, respectively [34]. These results demonstrate the importance of developing structurally distinct inhibitors to minimize the impact of drug-resistant mutations on inhibitor efficiency.

### 3.5. Macrodomain (Mac1)—nsp3

**Figure 4:**
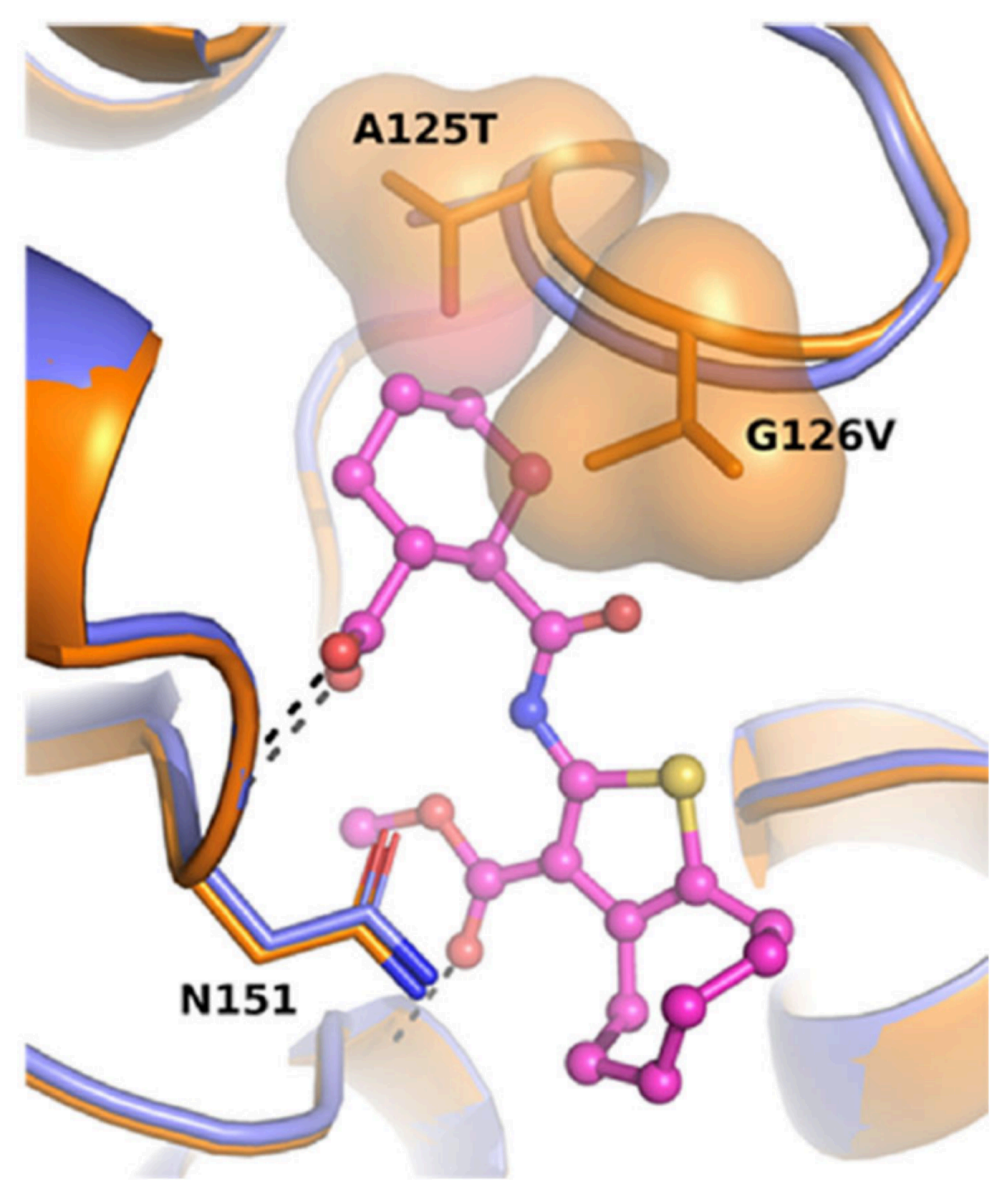
The structural model illustrates the drug resistance mutations in SAR-CoV-2 Mac1, selected for through the presence of compound **27**. The cocrystal structure of **27** bound to SARS-CoV-2 Mac1 was superimposed onto MHV Mac1. Models generated with Alphafold2 show WT (blue) and A125T/G126V mutant (orange) (residues based on Mac1 protein sequence and are equivalent to A1438T/G1439V described in the text) relative to the positions of the mutated residues and Asn151 (Phe in Mac1), which are shown as sticks and on the molecular surface. Image was obtained from Wazir et al. [43] without modification, https://pubs.acs.org/doi/10.1021/acs.jmedchem.3c02451.

The coronavirus conserved macrodomain is a small domain near the N-terminus of nsp3 and sits on the cytosolic side of the nsp3 pore [35]. Mac1 binds to ADP-ribose and has ADP-ribosylhydrolase activity. Its major role during infection is to counter host-mediated ADP-ribosylation [36]. Multiple studies have demonstrated that, in the absence of Mac1 activity, multiple CoVs are unable to cause disease in mice due to ADP-ribosylation-mediated restriction of viral replication and lead to enhanced interferon responses in both cell culture and in mice [37,38,39,40]. The extreme attenuation of these viruses has made Mac1 an attractive target for antiviral development, as multiple groups have carried out fragment and high-throughput screens for novel inhibitors of Mac1 biochemical activity [16]. Compound optimization has now led to several compounds with antiviral activity both in cell culture and in mice [41,42,43]. To confirm the specificity of two of these compounds, MDOLL-0229 (**27**) and MCD-814 (**5a**), our group performed viral passaging experiments using murine hepatitis virus (MHV) in the presence of these drugs until viral resistance was observed. Interestingly, in both cases, a double-mutant virus, A1438T/G1439V (residues numbers based on position in pp1a polyprotein) emerged, which provided at least partial resistance to a couple of Mac1 inhibitors [42,43]. Molecular modeling indicated that the valine residue protrudes into the ADP-ribose binding pocket to restrict the binding of compound **27** (Figure 4) [43]. Ironically, we had already engineered these mutations into a recombinant MHV as part of a separate project. We initially tried to produce a single G1439V MHV but were unable to recover G1439V without either reversion to WT or incorporating the A1438T mutation, indicating that strictly introducing the valine residue is detrimental for the virus likely by reducing ADP-ribose binding [44]. Introduction of the threonine residue appears to pull the valine residue slightly away from the ADP-ribose binding pocket while maintaining the ability to push out compound **27** from the binding pocket (Figure 4). MHV A1438T/G1439V replicated in cell culture, unlike G1439V, but had a significant replication defect in L929 cells. Furthermore, all mice survived infection with this virus in vivo, indicating that the virus is highly attenuated likely due to reduced replication [44]. Since this two-amino-acid mutation emerged separately following passaging of two distinct compounds, it appears that there are likely few paths available for Mac1 to develop resistance to inhibitors targeting this region of the protein. Furthermore, the fact that these mutations result in a virus that is highly attenuated in vivo and replicates poorly in cell culture suggests that a SARS-CoV-2 virus with similar mutations would be unlikely to cause disease in humans. However, further experiments with inhibitors that do not stretch into this region of Mac1 are needed to support this hypothesis.

## 4. Public Health Impact

The widespread use and potential misuse of antiviral drugs can lead to the emergence of resistant variants. Taking antiviral medication for extended periods of time, taking less than needed, or shortening treatment creates an environment in which these resistant strains develop. If these variants outcompete existing strains through increased transmission or replication, this could lead to rapid spread of the virus and create a major public health risk.

Chronic infection can arise from the persistent viral replication in immunocompromised patients acting as incubators [45]. The weakened immune system’s inability to clear the virus results in continuous viral replication [45]. This leads to more opportunities for viral mutation within a single host. The drug-resistant mutations E166A/V have been reported in immunocompromised patients undergoing treatment with Paxlovid [26]. Due to the lack of immune clearance, these patients have increased susceptibility to drug-resistant mutations.

Finally, there is a risk of drug-resistant mutants spreading through positive epistasis, where compensatory mutations allow a potent viral strain to dominate. A combination of mutations poses a public health threat as the virus may develop increased replication or transmission abilities. This can ultimately lead to treatment failure, which is especially detrimental to immunocompromised patients.

## 5. Anti-Resistance Strategies for COVID-19

As resistance has been reported for all three approved antivirals for COVID-19, measures need to be taken to prevent the spread of drug resistance and limit its impact in the clinic. First, it is important to properly manage drugs by only giving them to patients when they can be effective and ensuring that patients take the proper dose and entire course of antibiotics. Most antivirals are only effective if taken within a window of time from the onset of symptoms, and giving patients the drugs outside of that window will invite resistance to occur. Surveillance for drug resistance is also critical. Next-generation sequencing can be used on wastewater (including hospital wastewater) and clinical samples to identify known drug-resistant mutations of SARS-CoV-2 in the population and can help guide physicians to make decisions on therapeutic strategies [46,47]. SABRes is a bioinformatic tool that can detect drug-resistant mutations by utilizing expanding public datasets [48]. Surveillance will require knowledge on all potential drug-resistant mutations, so efforts should be made in research labs to identify potential mutations conferring resistance to these compounds and others that could be used in the clinic. However, developing drug-resistant mutations in the lab can be considered as gain-of-function research, and so special care and approaches will be needed, such as using highly attenuated variants of SARS-CoV-2 or non-human coronaviruses, such as murine hepatitis virus (MHV), when performing these experiments [42,49].

To try to prevent drug resistance altogether, a number of strategies can be employed, including combination therapy, novel inhibitor development guided by the envelope binding hypothesis, and drug design guided by structural models that avoid known hotspots of drug resistance. The classical example of combination therapy is in the HIV field, where anti-retroviral therapy (ART) has effectively thwarted drug resistance [50]. In HIV, 36 drugs have been approved, with most patients receiving the combination of an integrase inhibitor and a nucleoside analog. These treatments have provided incredible results in preventing HIV infection from developing into AIDS. In fact, most HIV patients now have life expectancies nearing that of the general population [50]. For influenza, combination therapies have not been successful in clinical trials, and so standard care is to treat with oseltamivir (Tamiflu^®^). However, resistance is monitored regularly through sequencing, and many other therapies are available, such as favipiravir (Avigan^®^), a nucleoside analog that targets PB1, and baloxavir marboxil (Xofluza^®^), which targets the endonuclease domain of PA [51]. The number of antivirals for influenza targeting multiple viral proteins is key to limiting drug resistance. Combination therapies have been tested for SARS-CoV-2 and several look promising at least in cell culture, animal models, and small studies in immunocompromised individuals. However, further clinical research with greater number of patients is needed to establish the benefit of combination therapy in humans [52].

The envelope binding method and structure-guided drug design are additional methods to avoid drug resistance. In the envelope binding method, inhibitors are designed that fit completely within the conserved binding pocket of the natural ligand (the “substrate envelope”). This method has been used to develop novel HIV-1 protease inhibitors that are less susceptible to drug-resistant mutations [53,54]. Structure-guided drug design should be utilized to target highly conserved regions of viral targets and avoid drug-resistant “hot spots”. Crystal structures exist for all of the SARS-CoV-2 non-structural protein targets described here, which can assist in further drug development plans, and structure-guided drug design has already been utilized to improve the initial screening of compounds or to identify new targets [55,56,57,58,59].

## 6. Conclusions

While there are effective SARS-CoV-2 antiviral therapies, the constant evolution of SARS-CoV-2 through point mutations and recombination creates variants that have mutations in common drug targets, most notably the viral polymerase (nsp12) and main protease (nsp5). The evolution of drug-resistant strains of SARS-CoV-2 emerges through selective pressures inherent in drug treatment. In some cases, these mutations reduce viral fitness or the ability of the virus to replicate or transmit between hosts. However, compensatory mutations can counteract this deficit by restoring or improving viral fitness, which can then threaten public health through untreatable variants. Of these two targets, far more drug-resistant mutations have been observed in Mpro, and one strategy to avoid resistance may be to transition to different protease inhibitors, such as moving from nirmatrelvir to ensitrelvir or other protease inhibitors at regular intervals.

There are many implications that antiviral resistance has for future drug design. As discussed above, future efforts could further explore incorporating a combination therapy approach with multiple antivirals, similar to the cocktail approach used to treat HIV. Viral proteins that antagonize host immune responses could be targeted, including PLpro, nsp15, or Mac1. These so-called “host-enabling” drugs could be effective alongside more-traditional drug targets. Other options will be to use rational drug design through structural biology to avoid hotspots of resistance, as described above. Additional drug development strategies targeting RNA species or utilizing proteolysis-targeting chimeras (PROTACs) also could limit the appearance of resistance.

Even with many advances in the development of effective antivirals, there is still concern about the drug-resistant evolution of SARS-CoV-2 variants due to the constant race between drug development and viral evolution. Efforts in the lab to identify novel mechanisms of drug resistance for viral targets will be key to understanding how these viral targets develop resistance. Then incorporating variant surveillance for drug-resistant viruses in wastewater and in patients will help guide specific treatment options going forward. Ultimately a strategy incorporating combination therapies, structurally guided drug design, strict treatment adherence, and continuous global surveillance can limit the impact of drug-resistant mutations on human disease caused by COVID-19.

## Figures and Tables

**Table 1:** SARS-CoV-2 antivirals and targets discussed in this review.

Antiviral	Brand Name	Target
Remdesivir	Veklury^®^	RdRp
Molnupiravir	Lagevrio^®^	RdRp
Nirmatrelvir	Paxlovid^®^	Mpro
Ensitrelvir	Xocova^®^	Mpro
Ibuzatrelvir	N/A	Mpro
Simnotrelvir	N/A	Mpro
Bofutrelvir	N/A	Mpro
EPB-113	N/A	EndoU
PF07957472	N/A	PLpro
Jun12682	N/A	PLpro
WEHI-P8	N/A	PLpro
MDOLL-0229	N/A	Mac1
MCD-814	N/A	Mac1

## Data Availability

No new data were created or analyzed in this study. Data sharing is not applicable to this article.
